# BFJPM ameliorates OVA-induced food allergy in a murine model accompanied by gut microbiota remodeling

**DOI:** 10.3389/fimmu.2026.1827925

**Published:** 2026-05-28

**Authors:** Xue Yang, Huiyi Peng, Rao Hu, Yifan Li, Ping Jiang

**Affiliations:** 1 The First Hospital of Hunan University of Chinese Medicine, Changsha, China; 2 Hunan University of Chinese Medicine, Changsha, China

**Keywords:** 16S rRNA sequencing, bufeijianpi mixture, food allergy, gut microbiota, network pharmacology

## Abstract

BufeiJianpi Mixture (BFJPM) is a modified herbal formula developed from long-term clinical practice for the management of allergic constitution in children. To evaluate the therapeutic efficacy of BFJPM in an OVA-induced murine model of food allergy and to investigate its effects on the gut microbiota through integrated animal experiments, 16S rRNA gene sequencing, and network pharmacology. Methods: Sixty 3-week-old female BALB/c mice were randomly assigned to six groups: negative control (NC), FA model, cetirizine, BFJPM-L, BFJPM-M, and BFJPM-H. An OVA-induced food allergy model was established. Therapeutic efficacy was assessed using allergy symptom scores, the V/C ratio of the small intestinal mucosa, and serum total IgE levels. Gut microbiota profiles were analyzed by 16S rRNA sequencing of intestinal contents. PICRUSt2 was used to predict microbial functional profiles, and network pharmacology was applied to identify key targets and pathways involved in BFJPM-mediated intervention. Results: BFJPM reduced allergy symptom scores and serum total IgE levels and improved the V/C ratio in FA mice. NMDS analysis showed significant differences in gut microbiota composition among groups (stress = 0.141). BFJPM increased microbial richness and altered community structure, with enrichment of *Actinobacteria* and *Candidatus Arthromitus*. Among the differential taxa, *Saccharopolyspora* emerged as the most promising genus associated with BFJPM intervention and was negatively correlated with serum IgE levels. Integrated network pharmacology and microbial functional prediction identified IL6, TNF, and IL10 as major targets of BFJPM in FA. The African trypanosomiasis pathway was identified as a shared pathway linking BFJPM, FA, and gut microbiota-associated functional alterations. Conclusion: BFJPM alleviated food allergy symptoms, reduced serum IgE levels, and improved intestinal mucosal integrity in OVA-induced FA mice. These effects were accompanied by remodeling of the gut microbiota. *Saccharopolyspora* may represent a microbial feature associated with BFJPM treatment. The African trypanosomiasis pathway may indicate a potential point of convergence between BFJPM-regulated host pathways and microbiota-associated functional changes. However, the causal relationship between microbiota remodeling and therapeutic efficacy remains to be established.

## 1 Introduction

FA is defined as an adverse immune reaction elicited by food protein antigens. According to the underlying immunopathological mechanisms, FA can be categorized as IgE-mediated, non-IgE-mediated, or mixed IgE/non-IgE-mediated. Among these subtypes, IgE-mediated FA is the most frequently encountered in clinical practice (1). A 20-year cross-sectional survey from Chongqing, China, reported a marked rise in pediatric food allergy prevalence, increasing from 3.5% in 1999 to 11.1% in 2019 (2). Current conventional approaches for FA management include allergen avoidance, oral immunotherapy, and biologic targeted therapies. However, overly restrictive avoidance diets may predispose affected children to nutritional deficiencies, malnutrition, or growth impairment. Oral immunotherapy typically requires a prolonged treatment course and often fails to confer sustained desensitization after discontinuation. In addition, the long-term safety and potential adverse effects of biologic therapies remain to be fully established (3). Accordingly, there is a clear need to advance and evaluate new approaches for FA prevention and management.

Growing evidence suggests that early-life disruption of the gut microbiota may increase susceptibility to FA in children. Compared with healthy children, those with FA often exhibit reduced microbial diversity (4). Mechanistically, the gut microbiota can modulate immune cell function to maintain immune homeostasis and reinforce intestinal barrier integrity, thereby suppressing allergic responses and offering a potential avenue for the prevention and management of IgE-mediated FA (5, 6). Professor Shulan, a nationally recognized senior TCM expert (seventh cohort), developed BFJPM on the basis of long-term clinical practice aimed at managing allergic constitution in children. BFJPM is a modified herbal formulation derived from two classical prescriptions, Liujunzi Decoction and Yupingfeng Powder, originally recorded in Taiping Huimin Heji Jufang and Jiuyuan Fang. It has been widely applied in the Department of Pediatrics at the First Affiliated Hospital of Hunan University of Chinese Medicine, especially for atopic children with the TCM pattern of lung–spleen dual deficiency. Preliminary studies indicate that BFJPM exerts therapeutic benefits in pediatric allergic disorders and reduces serum IgE and ECP levels in children with cough-variant asthma, thereby attenuating IgE-mediated hypersensitivity responses (7). Modern studies have further shown that the major active constituents of several herbs contained in BFJPM, including *Astragalus membranaceus*, *Atractylodes macrocephala*, *Pseudostellaria heterophylla*, and *Poria cocos*, possess the ability to regulate the gut microecology and ameliorate gastrointestinal diseases (8–11).

Given the intestinal localization of FA and the oral route of BFJPM administration, we included gut microbiota profiling to explore whether microbial alterations were associated with the therapeutic effects of BFJPM. In this study, we employed an OVA-induced murine model to evaluate the therapeutic efficacy of BFJPM and to characterize BFJPM-associated alterations in the gut microbiota. Specifically, we assessed treatment outcomes in FA mice following BFJPM administration and delineated the microbiota features associated with BFJPM intervention. In addition, we combined microbiome profiling with network pharmacology to probe microbiota-linked mechanisms that may underlie BFJPM-mediated protection against OVA-induced FA.

## 2 Materials and methods

### 2.1 Chemicals and reagents

Aluminum hydroxide (Shanghai Macklin Biochemical Co., Ltd.; Cat. No. A800852), ovalbumin (Shanghai Macklin Biochemical Co., Ltd.; Cat. No. E6337), and an IgE ELISA kit (Shanghai Kexing Biotechnology Co., Ltd.; Cat. No. A-M00055A) were used in this study.

### 2.2 Animals

Sixty 3-week-old female SPF BALB/c mice were purchased from Hunan Sileike Experimental Animal Co., Ltd. (Laboratory Animal Production License No. SCXK [Xiang] 2019-0004). The animals were maintained in the SPF animal facility of the First Affiliated Hospital of Hunan University of Chinese Medicine (Laboratory Animal Use License No. SYXK [Xiang] 2020-0010) and received standard chow from the university’s Experimental Animal Center. All experimental procedures were reviewed and approved by the Institutional Animal Care and Use Committee of the First Affiliated Hospital of Hunan University of Chinese Medicine (Approval No. 202404023).

### 2.3 Medicine

BFJPM was provided by the First Affiliated Hospital of Hunan University of Chinese Medicine and registered with the Hunan Provincial Medical Products Administration (Approval No. Z20210391000; dosage form: 100 ml per bottle). BFJPM comprises 11 herbal materials: *Pseudostellaria heterophylla* (Taizishen, 90g), *Astragalus membranaceus* or *A. membranaceus* var. *mongholicus* (Huangqi, 90 g); *Atractylodes macrocephala* (Baizhu, 50g), *Poria cocos* (Fuling, 90g), *Pinellia ternata* (Fabanxia, 30g), *Citrus reticulata pericarp* (Chenpi, 30g), *Saposhnikovia divaricata* (Fangfeng, 60g), *Schisandra chinensis fruit* (Wuweizi, 30g), *Hordeum vulgare* (Chaomaiya, 150g), *Gallus gallus domesticus gizzard inner lining* (Jineijin, 50g), and *Glycyrrhiza uralensis/G. inflata/G. glabra* (Zhigancao, 30g).

The OVA sensitization and challenge solutions were prepared according to the protocol reported by Meng Xiao et al. (12). For sensitization, we prepared a fresh injectable suspension of OVA and aluminum hydroxide in sterile normal saline (100 μg OVA and 1 mg aluminum hydroxide per 0.5 ml). For oral challenge, we freshly dissolved OVA in sterile normal saline to 2 mg/ml immediately before administration (12). Cetirizine hydrochloride drops (Bright Future Pharmaceuticals Factory; Approval No. JX20170375; 0.1 g/10 ml per bottle) were obtained from the First Affiliated Hospital of Hunan University of Chinese Medicine and diluted with distilled water to 0.0238 mg/ml. Cetirizine was used as a positive control because the OVA-induced FA model is characterized by IgE-mediated immediate hypersensitivity. Cetirizine is a clinically used second-generation H1 receptor antagonist with established anti-allergic symptom-relieving effects.

### 2.4 Establishment of ovalbumin-mediated food allergy and BFJPM treatment

The OVA-induced food allergy (FA) model adopted in this study is a well-established experimental model widely used to evaluate IgE-associated allergic responses and intestinal mucosal injury (13). After arrival, mice were acclimated to the animal facility for 7 days under specific pathogen-free (SPF) conditions before the initiation of the experiment. All experimental procedures were initiated when the mice were 4 weeks old. Briefly, mice in the model group received intraperitoneal injections of 0.5 ml sensitization solution containing 100 μg OVA and 1 mg aluminum hydroxide in normal saline on days 1, 7, and 14. Beginning on day 16, mice were orally challenged with a 10-fold sensitizing dose via gavage, receiving 0.5 ml OVA solution (2 mg/ml in normal saline) every other day (14). Allergic manifestations were monitored within 60min after each challenge, and symptom scores were recorded; a score of 1–3 was considered indicative of successful sensitization (12).

On experimental day 21, sensitized mice were randomly assigned to the FA model group, cetirizine group, BFJPM-L, BFJPM-M, or BFJPM-H group and received the indicated treatments by oral gavage. Doses were calculated using a body surface area–based interspecies conversion and corresponded to the daily pediatric dose for a 1-year-old child weighing 10kg. BFJPM was administered once daily at 40, 70, and 150 ml/kg in the BFJPM-L group, BFJPM-M group, and BFJPM-H groups, respectively, while the cetirizine group received 1.19 mg/kg/day. Mice in the negative control (NC) and FA model groups were administered 1 ml of normal saline by gavage once daily. All treatments were continued for 14 consecutive days (15). The NC group consisted of non-sensitized mice that received saline instead of OVA during both sensitization and challenge procedures and were administered saline by gavage to match the treatment protocol, serving as the baseline for comparison. The present study was conducted as a single experimental cohort with multiple biological replicates per group.

### 2.5 Clinical assessment of anti-food allergic effects of BFJPM

Clinical food allergy symptom scores and BMI were used as the primary outcome measures to monitor disease progression in OVA-mediated FA mice, and assessments were performed weekly. Symptom severity was evaluated using a previously reported murine FA scoring system (**Table 1**) (12). In addition, TCM syndrome manifestations were assessed according to the criteria described in *Diagnostics of Chinese Medical Syndromes* (16). On day 36, mice were anesthetized by intraperitoneal injection of pentobarbital sodium (50 mg/kg). Under deep anesthesia, euthanasia was confirmed by cervical dislocation, followed by immediate tissue collection. Serum IgE concentrations were subsequently determined using a commercial mouse IgE ELISA kit (Shanghai Kexing Biotechnology Co., Ltd., Shanghai, China; Cat. No. A-M00055A; standard curve range, 7.5–240 ng/ml).

**Table 1:** Clinical scoring system for FA in Murine models.

Severity score	Degree of FA
0	No clinical symptoms were observed.
1	Mice demonstrated repetitive grooming behaviors targeting oral, nasal, and aural regions, with a defined threshold of >10 instances per observation period.
2	Reduced locomotor activity; increased incidence of isolated movement patterns; evident erythema and edema in both auricular and periocular regions; nasal flaring; persistent diarrhea
3	Prolonged immobility (>1 min duration); tachypnea with audible wheezing and dyspnea; alopecia with concomitant perinasal and snout erythema and edema; cyanosis of oral mucosa and distal tail; maculopapular eruptions on both oral and caudal regions
4	Bilateral exophthalmos; conjunctival hyperemia; unresponsiveness to external stimuli; intermittent myoclonic jerks or high-amplitude tremors
5	Generalized tremors; shock; death

### 2.6 Histopathological assessment of anti-food allergic effects of BFJPM

Euthanasia was confirmed by cervical dislocation under deep anesthesia, followed by immediate collection of small intestinal tissues. Samples were fixed in 4% paraformaldehyde for 24h, processed for paraffin embedding, and sectioned at a thickness of 5 μm. The sections were then baked at 60 C, dewaxed in an automated staining system, and stained with H&E for morphological assessment. Intestinal injury was graded using a modified Chiu’s scoring system (17, 18): (0) normal mucosal architecture; (1) widening of the subepithelial space at villus tips; (2) epithelial lifting from the lamina propria with expansion of the subepithelial space; (3) extensive epithelial lifting with bilateral villus tilting and partial denudation at villus tips; (4) denudation of villus lamina propria with dilation of exposed capillaries and increased cellularity in the lamina propria; and (5) degeneration or digestion of the lamina propria with hemorrhage and/or ulceration. Brightfield slide scanning was conducted by Hubei Biossci Biotechnology Co., Ltd. (Wuhan, China). Villus height and crypt depth were quantified using SlideViewer (Leica), and the resulting ratio was used as an indicator of mucosal integrity.

### 2.7 Gut microbiota analysis of anti-food allergic effects of BFJPM

Following euthanasia, we immediately dissected the mice to isolate the entire small intestine. We then gently scraped the luminal surface to collect intestinal contents. All samples were rapidly frozen and transported to Personal Biotechnology Co., Ltd. for subsequent analyses. Detailed procedures were conducted according to established protocols described previously (19).

To characterize the gut microbiota, we conducted 16S rRNA gene sequencing. Total genomic DNA was isolated from intestinal contents, and the V3–V4 hypervariable region of the bacterial 16S rRNA gene was amplified by PCR using universal primers (F: ACTCCTACGGGAGGCAGCA; R: GGACTACHVGGGTWTCTAAT). The resulting amplicons were purified, quantified by Qubit fluorometry, and sequenced on an Illumina MiSeq/NovaSeq platform by Personal Biotechnology Co., Ltd. Raw reads were processed in QIIME2 using DADA2 for denoising and generation of amplicon sequence variants, and the resulting data were used for taxonomic assignment as well as α- and β-diversity analyses. Using the 16S rRNA datasets, we employed PICRUSt2 to infer the functional potential of the microbial communities. Predicted gene families were annotated against the KEGG database to obtain KOs and pathway-level profiles, and we further assessed taxon-specific contributions to enriched pathways to identify the microbial taxa most strongly associated with key functional shifts (20). The raw sequencing data have been deposited in the NCBI Sequence Read Archive under BioProject accession number PRJNA1309614.

### 2.8 Network pharmacology analyses

Active constituents and corresponding targets for the major components of BFJPM (Huang Qi, Fang Feng, Bai Zhu, Ban Xia, Chen Pi, Fu Ling, Mai Ya, Wu Wei Zi, and Tai Zi Shen) were collected from TCMSP. Compounds were filtered using OB ≥ 30% and DL ≥ 0.18. For Zhigancao and Jineijin, candidate constituents and putative targets were retrieved from BATMAN-TCM with a score ≥ 30 and P ≤ 0.05. After removing duplicate entries, all targets were mapped to standardized gene symbols using the UniProt database to generate the putative BFJPM target set.

FA-related targets were collected from GeneCards using “food allergy” as the search term, with a relevance score threshold of ≥ 3.76; duplicates were removed to obtain a nonredundant set of disease-associated targets. Overlapping targets between BFJPM and FA were identified by Venn analysis. The intersecting genes were imported into STRING (v11.5) to construct a PPI network, with *Homo sapiens* selected as the reference species and a confidence score > 0.9. The resulting network was visualized in Cytoscape (v3.9.1). GO and KEGG enrichment analyses were subsequently conducted in DAVID (v6.8) using *Homo sapiens* as the background, and the results were visualized in RStudio (21).

### 2.9 Statistical analyses

Statistical analyses were performed in GraphPad Prism (v10.0) and SPSS (v25.0). Continuous data are expressed as mean ± SD. Normality was evaluated using the Shapiro–Wilk test. For normally distributed data with equal variances, group differences were assessed by one-way ANOVA followed by the LSD *post hoc* test. When variances were unequal, Dunnett’s T3 test was used. Non-normally distributed data were analyzed with the Kruskal–Wallis H test. Diagnostic performance was evaluated using ROC curve analysis.

## 3 Results

### 3.1 BFJPM significantly inhibits disease progression of FA mice

**Figure 1:**
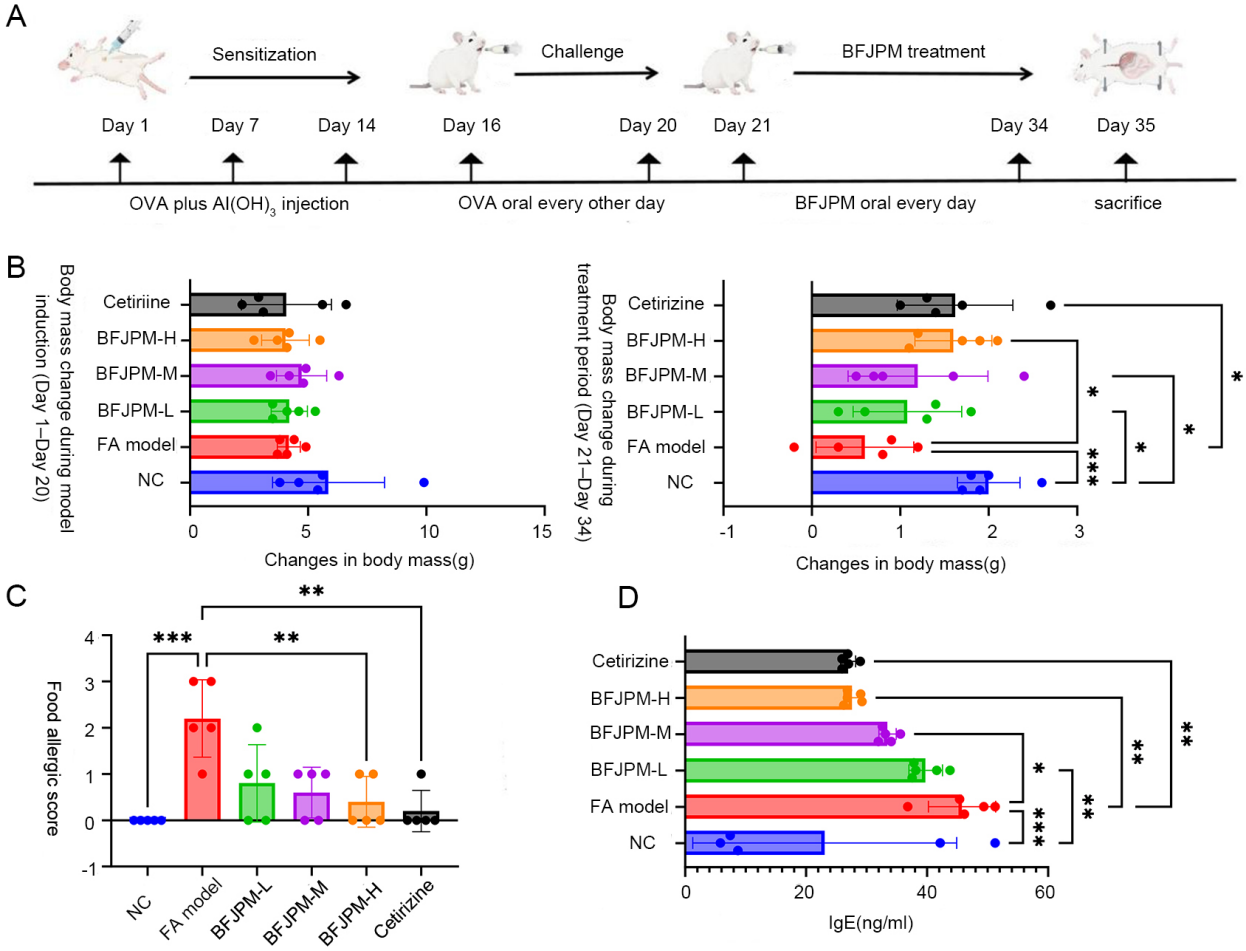
Anti-allergic effects of BFJPM in OVA-induced FA mice. **(A)** Schematic diagram of OVA induction and the BFJPM treatment protocol. **(B)** Body mass change during model induction (Day 1–Day 20) and during the treatment period (Day 21–Day 34). Body mass change during model induction was calculated as body mass on Day 20 minus body mass on Day 1, and body mass change during the treatment period was calculated as body mass on Day 34 minus body mass on Day 21. Individual data points are shown. **(C)** Food allergy scores in mice. **(D)** Quantitative analysis of serum IgE levels. *n*=5 per group. *P<0.05, **P<0.01, ***P<0.001, and ****P<0.0001.

Beginning on experimental day 21, mice received once-daily oral gavage of BFJPM at 40, 70, or 150 ml/kg through day 34 (**Figure 1A**). During the sensitization phase, FA model mice exhibited impaired weight gain (*p*<0.05) and increased allergy symptom scores (*p*<0.01) compared with controls (**Figures 1B, C**). BFJPM treatment ameliorated these clinical manifestations to varying extents. Consistently, serum IgE levels were markedly elevated in FA mice, showing a 4.6-fold increase relative to controls (*p*<0.0001; **Figure 1D**). BFJPM produced a dose-dependent reduction in serum IgE, with decreases of 13% at 40 ml/kg (*p*<0.01), 27% at 70 ml/kg (*p*<0.001), and 39% at 150 ml/kg (*p*<0.0001) (**Figure 1D**). Moreover, the effect of BFJPM-H was comparable to that of the cetirizine positive control (*p* > 0.05; **Figure 1D**). Taken together, these results demonstrate a clear dose–response relationship and indicate that the highest BFJPM dose achieves an anti-allergic effect similar to that of cetirizine.

### 3.2 BFJPM preserves intestinal mucosal integrity and restores the V/C ratio in FA mice

**Figure 2:**
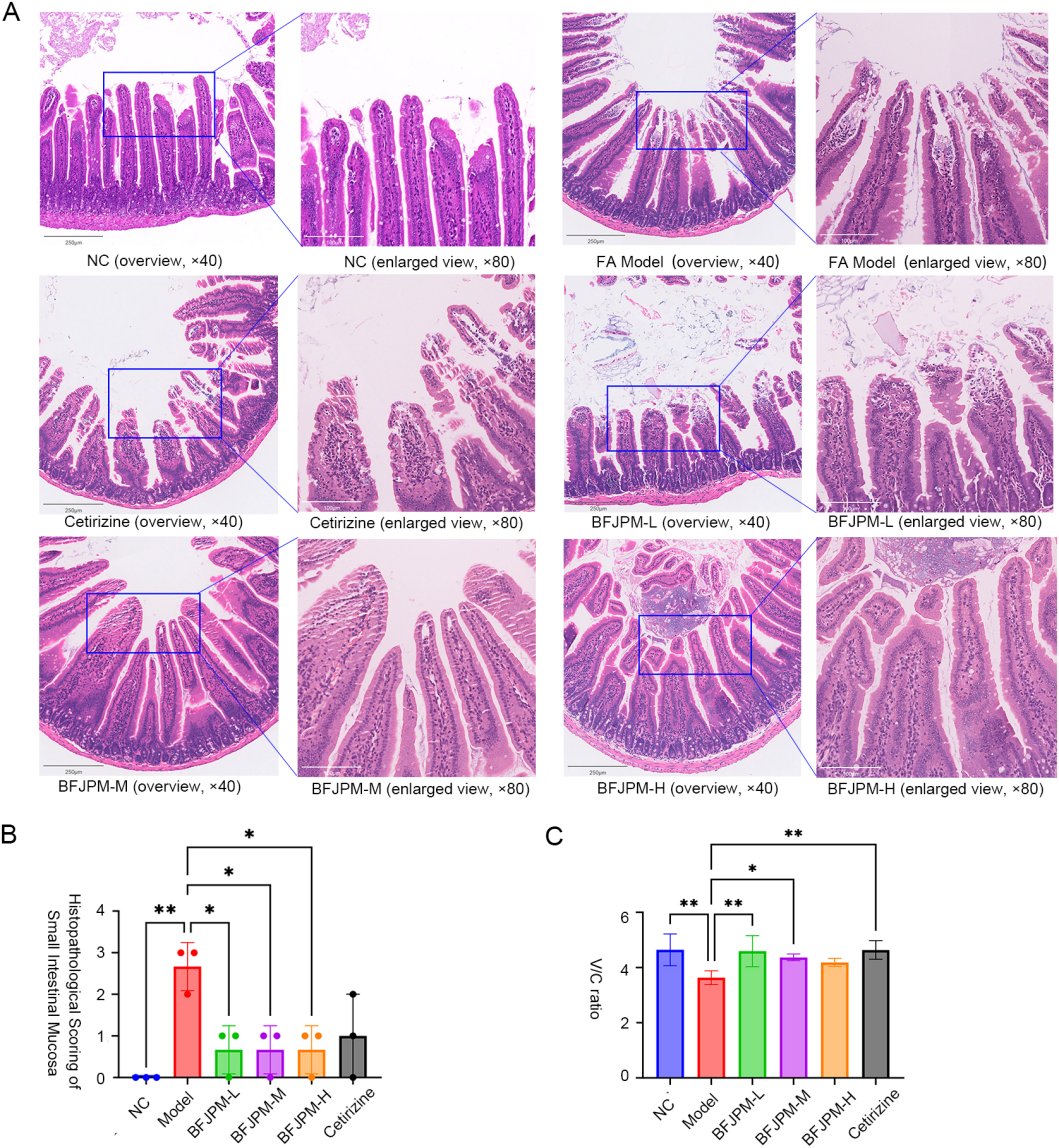
Protective effects of BufeiJianpi Mixture on small intestinal histopathology in ovalbumin-induced food allergy mice. **(A)** Representative hematoxylin and eosin-stained images of small intestinal sections. Overview images are shown at ×40 magnification, with boxed regions indicating the areas selected for enlarged views at ×80 magnification. Scale bars = 250 μm for ×40 images and 100 μm for ×80 images; *n*=3 per group. **(B)** Histopathological scores of the small intestinal mucosa (*n* = 3 per group). **(C)** Villus-to-crypt ratio of the small intestinal mucosa (*n* = 3 per group). *p<0.05, **p<0.01.

To examine the protective effect of BFJPM on FA-associated intestinal injury, we performed H&E staining of small intestinal tissues. In the NC group, sections showed preserved mucosal architecture with intact villi and basement membranes, and neatly arranged columnar epithelial cells with typical morphology (**Figures 2A, B**). Crypt structures were relatively shallow and clearly demarcated, and no evident pathological abnormalities were detected (**Figures 2A, B**). In contrast, the FA model group showed marked histopathological abnormalities, including villus disorganization, epithelial shedding at villus tips, and intestinal gland hyperplasia (**Figures 2A, B**).

In line with these morphological changes, the FA model group exhibited a significantly reduced V/C ratio relative to the NC group (*p*<0.05) (**Figure 2C**). Cetirizine, BFJPM-L, and BFJPM-H significantly increased the V/C ratio relative to the model group (*p*<0.05 and *p*<0.01) (**Figure 2C**). Collectively, these results indicate that OVA-induced FA induces intestinal mucosal damage accompanied by a reduced V/C ratio, whereas BFJPM partially reverses these changes, thereby preserving mucosal integrity and supporting barrier function.

### 3.3 BFJPM altered the gut content microbiota in FA mice

**Figure 3:**
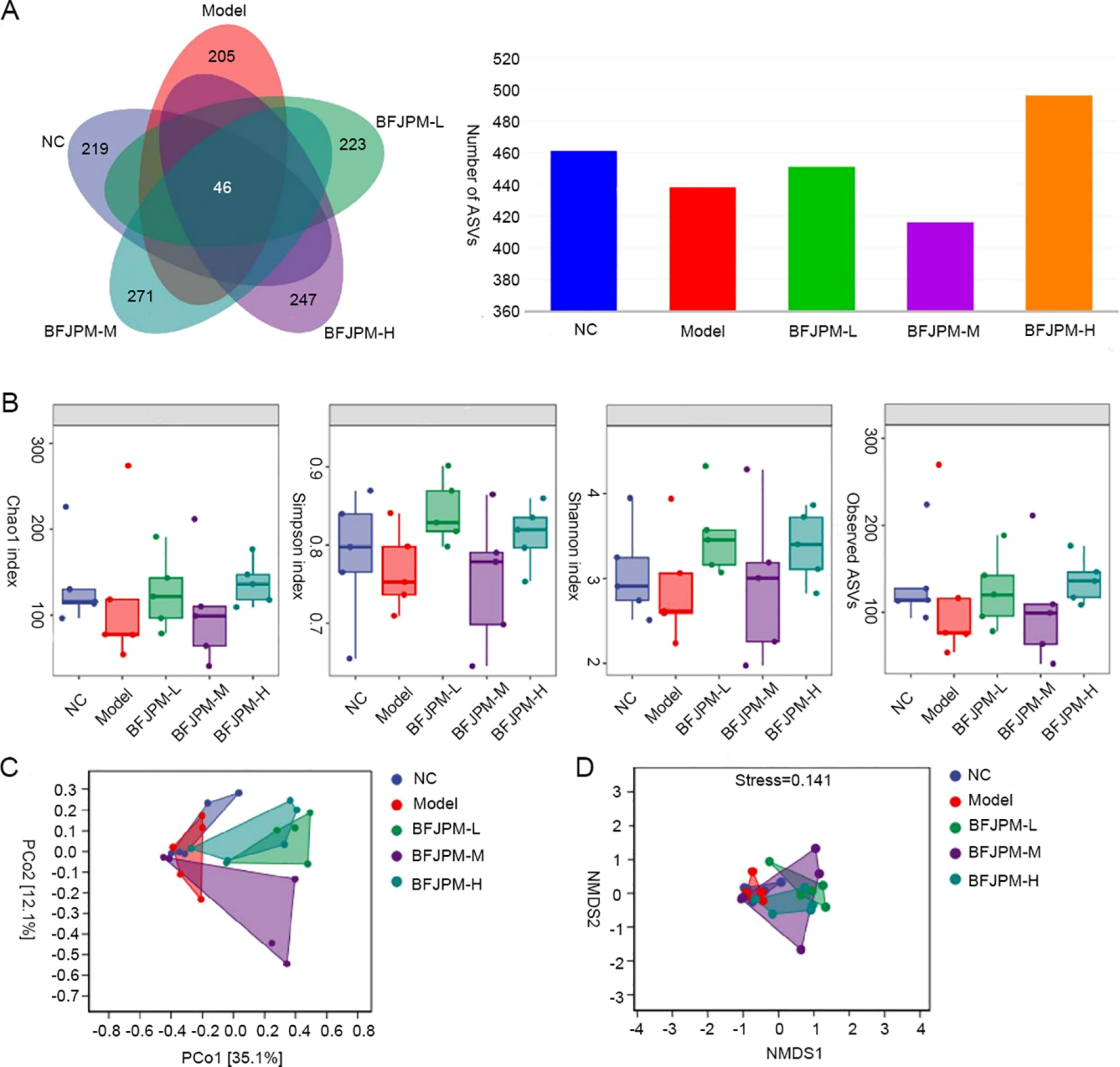
BFJPM reshapes gut microbial richness, diversity, and community structure in FA mice. **(A)** Venn diagram and bar plot showing shared and unique ASVs among groups. **(B)** α-diversity analysis of gut microbiota based on the Chao1, Simpson, Shannon, and observed species indices. **(C)** Principal coordinates analysis based on Bray–Curtis distances showing differences in microbial community structure among groups. **(D)** Non-metric multidimensional scaling analysis showing overall dissimilarity in gut microbiota composition among groups. These analyses were performed to evaluate whether BFJPM altered gut microbial richness, diversity, and community structure in FA mice. *n*=5 per group.

Gut microbiota profiling identified 46 ASVs shared across all groups (**Figure 3A**). The NC group contained 219 unique ASVs, whereas the model group contained 205. Notably, the model group exhibited the lowest ASV richness among all groups (**Figure 3A**). BFJPM treatment increased ASV abundance, and ordination analyses suggested clear differences in community composition among groups (**Figure 3A**).

Alpha diversity was evaluated using the Chao1, observed species, Shannon, and Simpson indices. Compared with the NC group, the model group showed marked declines in both microbial richness and evenness (**Figure 3B**). BFJPM altered microbial richness and diversity across treatment groups; however, these changes did not follow a strictly linear dose-response pattern, with BFJPM-L showing the most prominent improvement in α-diversity indices (**Figure 3B**). In Bray–Curtis-based PCoA, PCo1 and PCo2 explained 47.2% and 12.1% of the variance, respectively (**Figure 3C**). NMDS analysis yielded a stress value of 0.141, indicating that the ordination reliably reflected differences in community structure (**Figure 3D**). Both ordination methods showed clear separation among groups. Moreover, BFJPM treatment produced an evident shift in gut microbial composition relative to both the NC and model groups (**Figures 3C, D**). Collectively, these findings indicate that BFJPM reshapes the gut microbial ecosystem in FA mice.

### 3.4 Effects of BFJPM on dominant gut microbiota in FA mice

**Figure 4:**
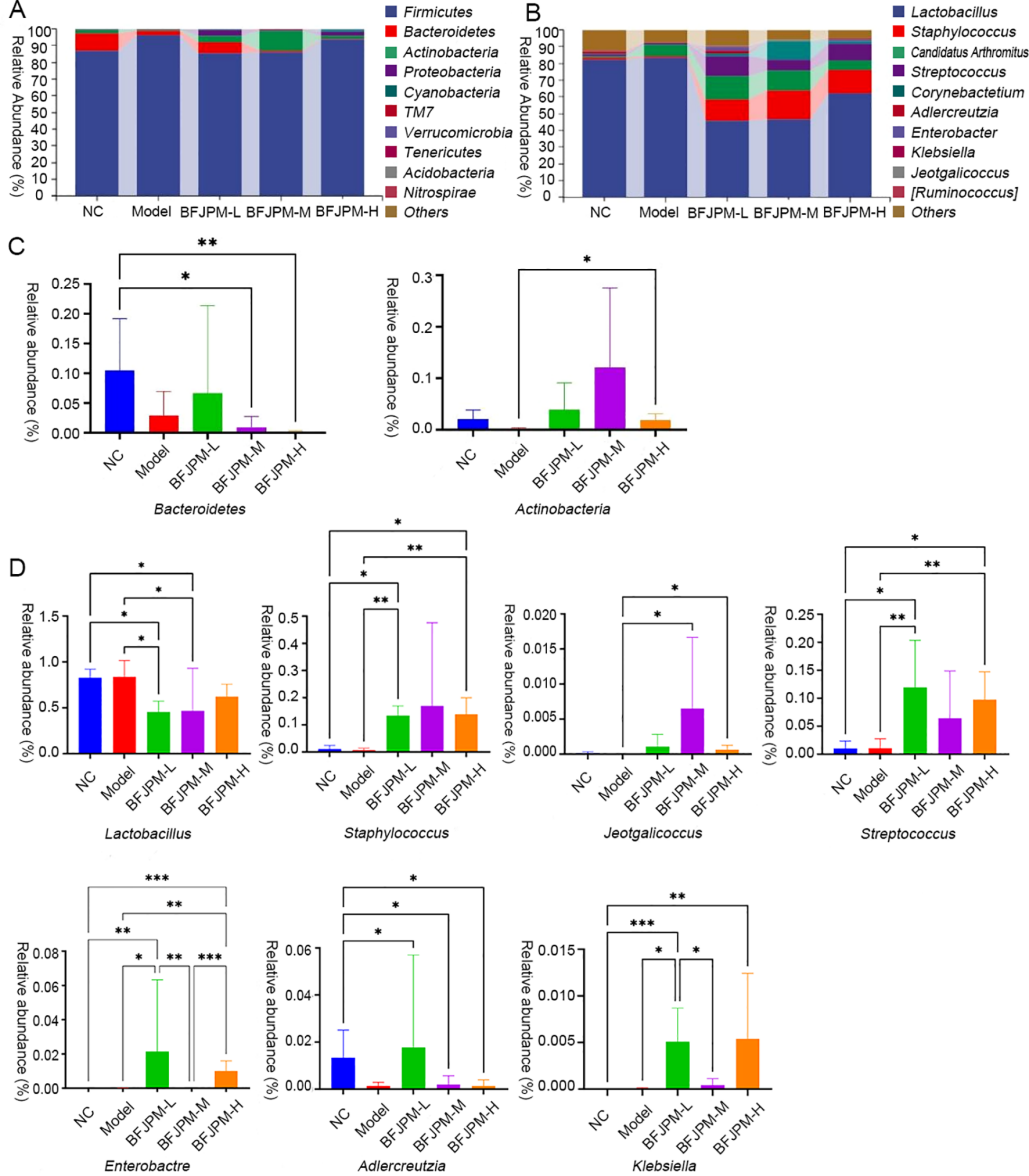
Analysis of the dominant bacteria in the gut contents of FA mice. **(A)** Phylum-level community composition and relative abundance. **(B)** Genus-level community composition and relative abundance. **(C)** Statistical analysis of relative abundance differences among the dominant gut microbiota phyla. **(D)** Statistical analysis of relative abundance differences among the top 10 bacterial genera. *n* = 5 per group. *P < 0.05, **P < 0.01, ***P < 0.001.

At the phylum level, *Firmicutes*, *Bacteroidetes*, and *Actinobacteria* were the dominant taxa, each accounting for >1% of the total community (**Figure 4A**). BFJPM induced dose-dependent changes in phylum-level composition. Compared with the model group, the relative abundance of *Firmicutes* decreased by 10.65%, 10.04%, and 2.22% in the BFJPM-L, BFJPM-M, and BFJPM-H groups, respectively, although these differences were not statistically significant (all *p* > 0.05) (**Figures 4A, C**). In contrast, BFJPM significantly reduced *Bacteroidetes* abundance in the BFJPM-M and BFJPM-H groups by 1.98% (*p*<0.05) and 2.74% (*p*<0.01), respectively (**Figure 4A, C**). *Actinobacteria* increased across all BFJPM-treated groups, with elevations of 3.62% (BFJPM-L), 12.65% (BFJPM-M; *p*<0.05), and 1.63% (BFJPM-H) (**Figures 4A, C**).

At the genus level, five genera—*Lactobacillus*, *Staphylococcus*, *Candidatus Arthromitus*, *Streptococcus*, and *Corynebacterium*—dominated the gut microbiota, each with a relative abundance >1% (**Figure 4B**). Analysis of the top 10 genera revealed marked BFJPM-related alterations. Specifically, *Lactobacillus* abundance was significantly lower in the BFJPM-L and BFJPM-H groups than in both the NC and model groups (*p*<0.05) (**Figure 4D**). *Adlercreutzia* also showed a significant decline across all BFJPM-treated groups relative to the NC group (*p*<0.05) (**Figure 4D**). By contrast, several genera were enriched after BFJPM treatment. *Staphylococcus*, *Streptococcus*, *Enterobacter*, and *Klebsiella* were all significantly increased in the BFJPM-L and BFJPM-H groups compared with both the NC and model groups (*p*<0.05) (**Figure 4D**). *Jeotgalicoccus* exhibited a dose-dependent upward trend, reaching significance in the BFJPM-M and BFJPM-H groups versus the NC group (**Figure 4D**).

After BFJPM administration, the relative abundance of *Lactobacillus*, a commonly recognized beneficial gut genus, decreased, whereas *Bacteroidetes* showed no marked change and *Actinobacteria* increased. This pattern suggests that BFJPM may competitively inhibit *Lactobacillus* colonization without depleting *Bacteroidetes*, while selectively enriching *Actinobacteria*, a phylum that includes taxa with antimicrobial properties. Such shifts may reflect, at least in part, the antimicrobial activity of BFJPM. In addition, the increased abundances of *Staphylococcus*, *Streptococcus*, *Enterobacter*, *Klebsiella*, and *Jeotgalicoccus*—often considered commensal or conditionally commensal taxa—indicate that BFJPM treatment reshaped the intestinal microbial community and broadened the gut microecological landscape in mice.

### 3.5 Effects of BFJPM on characteristic gut microbiota in FA mice

**Figure 5:**
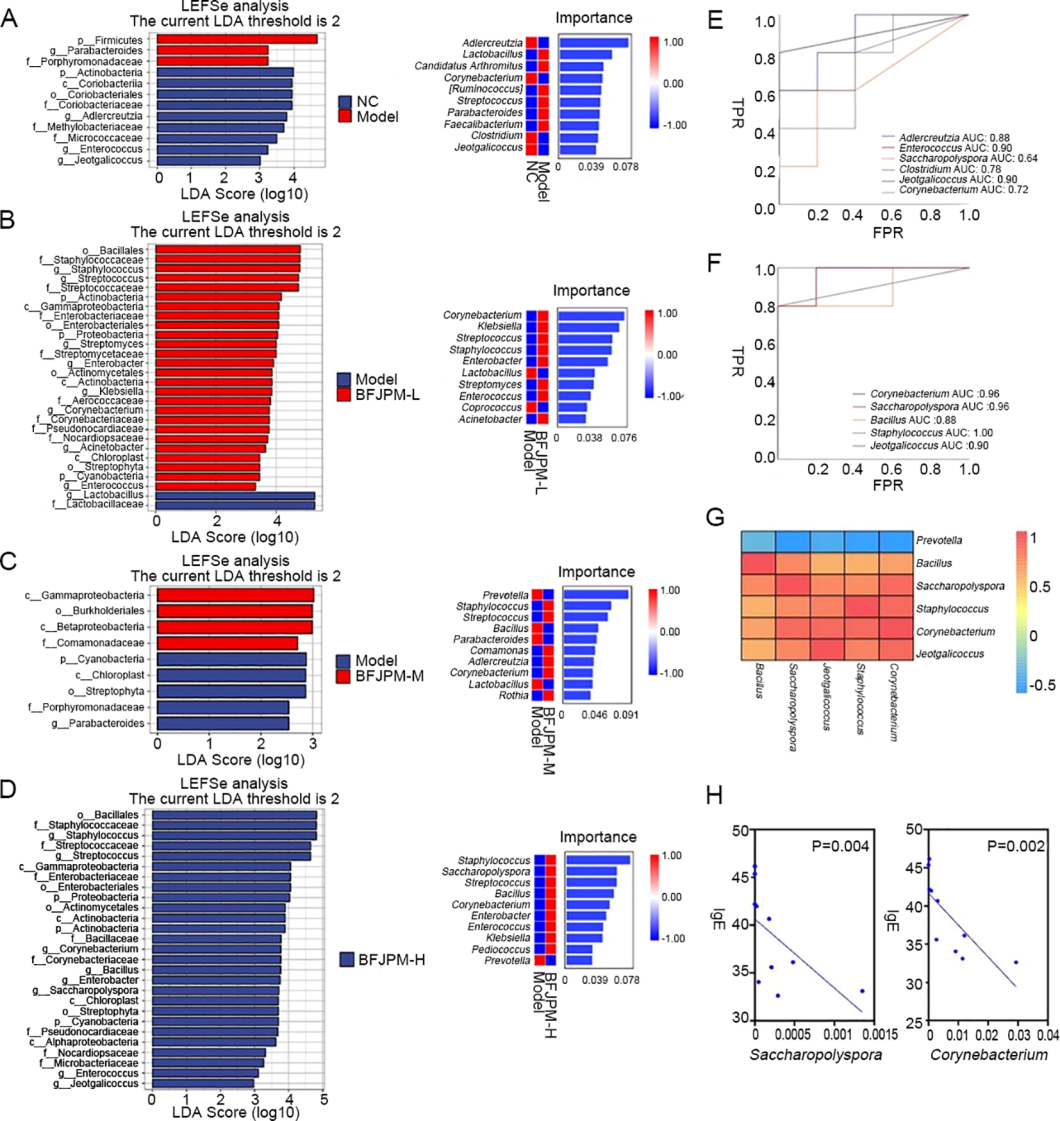
Identification and evaluation of characteristic bacterial taxa in the gut microbiota of food allergy mice. **(A–D)** Differential bacterial taxa identified by LEfSe analysis and Random Forest classification in pairwise comparisons between groups: negative control versus model **(A)**, BFJPM-L versus model **(B)**, BFJPM-M versus model **(C)**, and BFJPM-H versus model **(D)**. **(E, F)** Receiver operating characteristic (ROC) curve analysis of selected bacterial taxa for discriminating between negative control and model groups **(E)**, and between BFJPM-H and model groups **(F)**. Area under the curve (AUC) values indicate classification performance. **(G)** Correlation analysis of differential bacterial genera between the model and BFJPM-H groups. **(H)** Spearman correlation analysis between the relative abundance of Saccharopolyspora and Corynebacterium and serum IgE levels. LEfSe analysis was performed to identify differentially abundant taxa, and Random Forest was used to evaluate feature importance. Correlation analyses were conducted using Spearman’s rank correlation. Data are presented as mean ± SD (*n* = 5 per group).

To identify group-specific microbial signatures, we performed both LEfSe and Random Forest analyses. Relative to the model group, the NC group was enriched in *Adlercreutzia*, *Corynebacterium*, *Clostridium*, *Saccharopolyspora*, *Jeotgalicoccus*, and *Enterococcus* (**Figure 5A**). In contrast, the model group showed higher abundances of *Candidatus Arthromitus*, *Lactobacillus*, *Ruminococcus*, *Streptococcus*, *Parabacteroides*, *Faecalibacterium*, and *Klebsiella* (**Figure 5A**).

Relative to the BFJPM-L group, the model group exhibited higher abundances of *Lactobacillus* and *Coprococcus* (**Figure 5B**). In contrast, the BFJPM-L group was enriched in *Corynebacterium*, *Streptomyces*, *Streptococcus*, *Staphylococcus*, *Enterococcus*, *Enterobacter*, *Acinetobacter*, and *Klebsiella* (**Figure 5B**). Compared with the BFJPM-M group, the model group was enriched in *Parabacteroides*, *Prevotella*, *Bacillus*, and *Lactobacillus* (**Figure 5C**). Conversely, the BFJPM-M group showed higher abundances of *Staphylococcus*, *Streptococcus*, *Comamonas*, *Adlercreutzia*, *Corynebacterium*, and *Rothia* (**Figure 5C**). Relative to the BFJPM-H group, the model group exhibited a higher abundance of *Prevotella* (**Figure 5D**). In contrast, the BFJPM-H group was enriched in multiple genera, including *Corynebacterium*, *Saccharopolyspora*, *Bacillus*, *Jeotgalicoccus*, *Staphylococcus*, *Enterococcus*, *Streptococcus*, *Enterobacter*, *Klebsiella*, and *Pediococcus* (**Figure 5D**).

To identify gut microbial genera with the greatest potential to reflect BFJPM-mediated intervention in FA, we performed ROC analyses of the five most abundant genera in the NC and BFJPM-H groups. Genera with an AUC > 0.8 were considered to have good discriminative performance. *Adlercreutzia*, *Jeotgalicoccus*, and *Enterococcus* effectively distinguished the NC group from the model group (**Figure 5E**). In addition, *Saccharopolyspora*, *Corynebacterium*, *Bacillus*, *Jeotgalicoccus*, and *Staphylococcus* exhibited strong diagnostic potential for evaluating BFJPM efficacy in ameliorating FA (**Figure 5F**).

Correlation analysis of characteristic genera identified between the model and BFJPM-H groups revealed an overall inverse association, supporting the notion that BFJPM reshapes gut microbial community structure in FA mice (**Figure 5G**). Using a stringent threshold (|r| > 0.8), we further examined co-occurrence patterns within the BFJPM-H group and observed a strong positive correlation between *Saccharopolyspora* and *Corynebacterium* (**Figure 5G**). In contrast, *Staphylococcus* did not show significant correlations with other genera under the same criteria (**Figure 5G**). To explore potential links between microbial shifts and allergic responses, we performed Spearman correlation analysis between representative genera and serum IgE levels. *Saccharopolyspora* and *Corynebacterium* showed significant negative correlations with IgE in FA mice (**Figure 5H**), suggesting that enrichment of these taxa may be associated with reduced IgE levels and may contribute to the anti-allergic effects of BFJPM.

### 3.6 Network pharmacology analysis of BFJPM in food allergy

**Figure 6:**
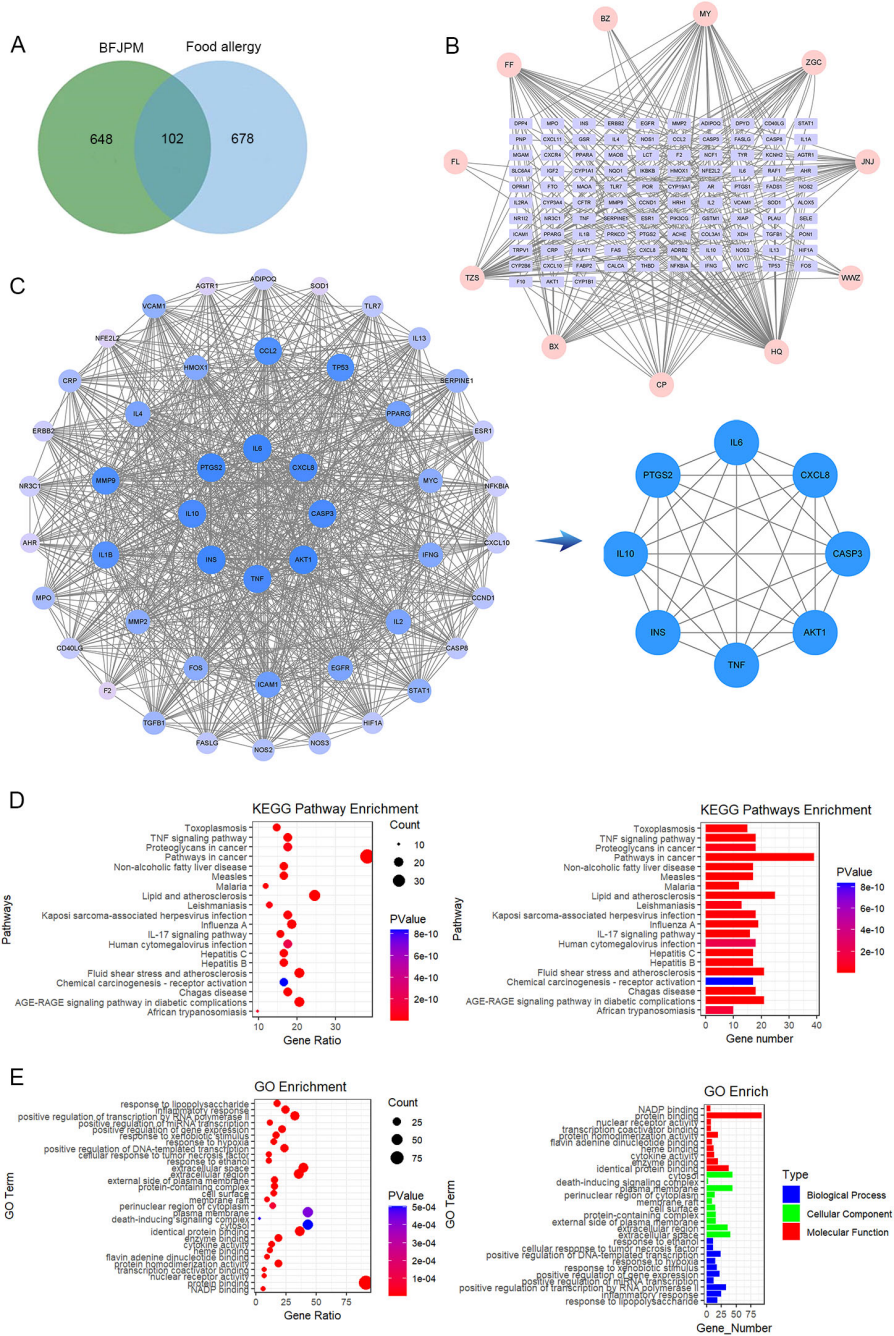
Network pharmacological analysis of BFJPM in FA mice. **(A)** Venn diagram of intersecting targets between BFJPM and FA. **(B)** Relationship between common targets and individual herbal constituents of BFJPM. **(C)** Screening of key targets via the PPI network. **(D)** KEGG pathway enrichment analysis. **(E)** Functional enrichment analysis of GO.

In the network pharmacology analysis, we first identified putative BFJPM targets based on its active ingredients and intersected them with FA-related targets using a Venn diagram, which yielded 102 shared targets (**Figure 6A**). The relationships between BFJPM compounds and these common targets are shown in **Figure 6B**. We then imported the overlapping targets into the STRING database to construct a PPI network (**Figure 6C**). Applying the topological thresholds of Degree > 20, Betweenness > 0.1, and Closeness > 0.5 generated a subnetwork containing 48 nodes and 850 edges. Subsequent screening with a more stringent Degree cutoff (Degree ≥ 45) identified eight hub targets—AKT1, INS, IL6, PTGS2, TNF, CASP3, IL10, and CXCL8—suggesting their central roles in BFJPM-mediated regulation of FA-related pathways (**Figure 6C**).

For GO enrichment analysis, the top 10 significantly enriched terms from the biological process, cellular component, and molecular function categories were selected for visualization (**Figure 6E**). KEGG pathway analysis identified the top 20 enriched pathways, including “Pathways in cancer, ” “AGE–RAGE signaling pathway in diabetic complications, ” “Lipid and atherosclerosis, ” and “Fluid shear stress and atherosclerosis” (**Figure 6D**). Additional pathways with potential immunological relevance included the “TNF signaling pathway, ” “IL-17 signaling pathway, ” “Influenza A, ” “Measles, ” “Malaria, ” and “African trypanosomiasis” (**Figure 6D**).

### 3.7 Prediction of metabolic pathways and functional profiles of gut microbiota in FA mice

**Figure 7:**
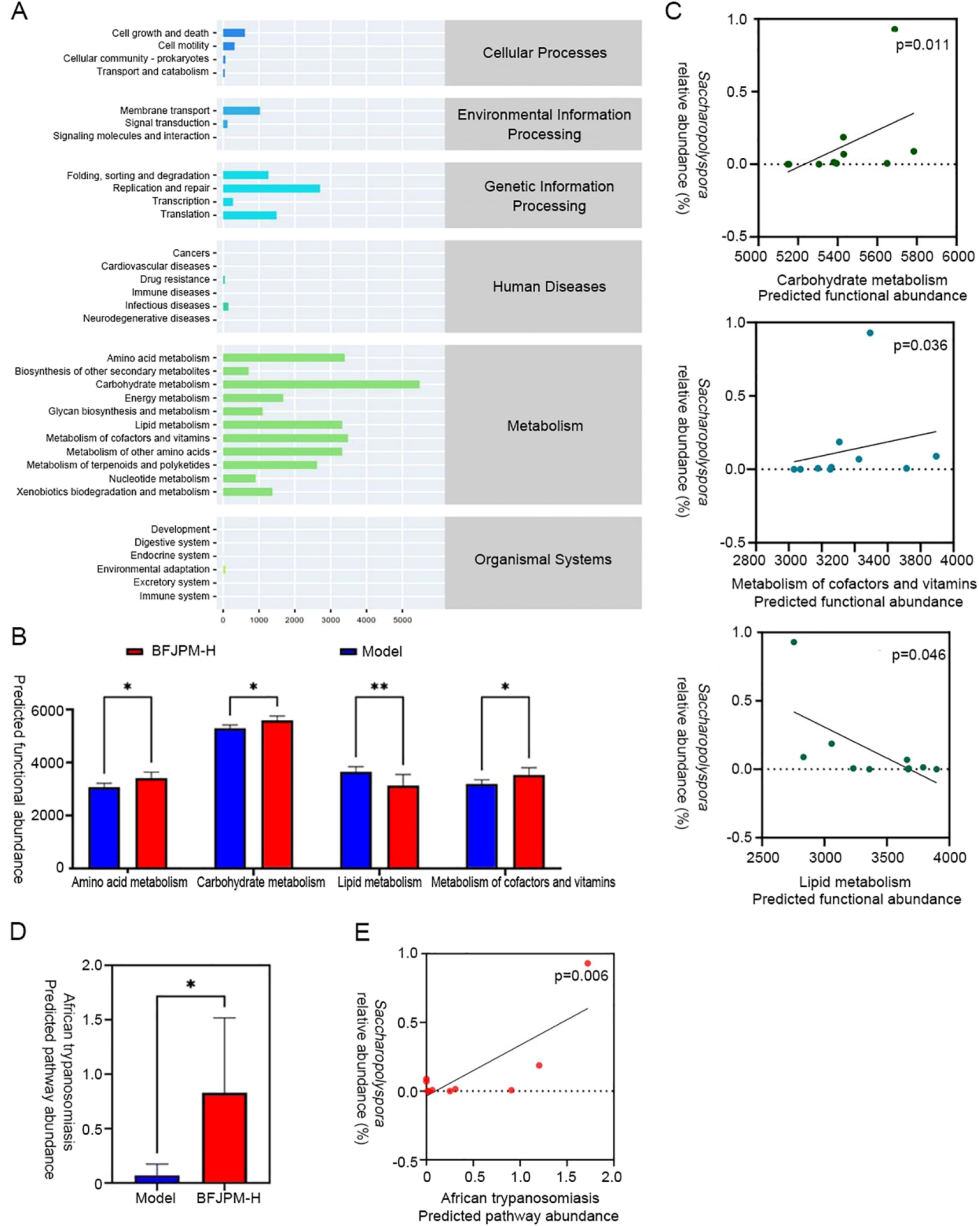
Prediction of metabolic pathways and functional profiles of the gut microbiota in food allergy mice. **(A)** Predicted KEGG functional profiles of the gut microbiota at different hierarchical levels. **(B)** Differential metabolic functions between the BFJPM-H and model groups. **(C)** Correlation analysis between *Saccharopolyspora* abundance and selected metabolic functions, including carbohydrate metabolism, metabolism of cofactors and vitamins, and lipid metabolism. **(D)** Predicted abundance of the African trypanosomiasis pathway in the BFJPM-H and model groups. **(E)** Correlation analysis between *Saccharopolyspora* abundance and the African trypanosomiasis pathway. Functional predictions were generated using PICRUSt2. Correlation analyses were performed using Spearman’s rank correlation. Data are presented as mean ± SD (*n* = 5 per group). *p < 0.05, **p < 0.01.

KEGG-based functional prediction of the gut microbial community identified six major functional categories at KEGG level 1. At level 2, 34 subcategories were detected, with metabolism accounting for a substantial proportion of the predicted functional repertoire (**Figure 7A**). Comparative analysis revealed significant differences between the model and BFJPM-H groups in four metabolism-related pathways: carbohydrate metabolism, lipid metabolism, metabolism of cofactors and vitamins, and amino acid metabolism (**Figure 7B**). *Saccharopolyspora* abundance was positively correlated with carbohydrate metabolism and metabolism of cofactors and vitamins, negatively correlated with lipid metabolism, and showed no significant association with amino acid metabolism (**Figure 7C**).

KEGG pathway profiling of the predicted gut microbiome functions identified 173 pathways in FA mice. Among the top 20 pathways showing the greatest responsiveness to BFJPM treatment, ko05143 (African trypanosomiasis) and ko05145 (toxoplasmosis) ranked highest. Notably, ko05143 showed significant enrichment in the BFJPM group compared with the NC group (**Figure 7D**). Moreover, Saccharopolyspora abundance was strongly positively correlated with the African trypanosomiasis pathway (**Figure 7E**), suggesting that this pathway may constitute a key mechanistic node linking BFJPM-associated microbial shifts with FA-related immune modulation.

## 4 Discussion

### 4.1 Therapeutic efficacy of BFJPM in OVA-induced FA mice

FA poses a diagnostic challenge because of its heterogeneous clinical manifestations, which frequently overlap with those of other disorders. Typical presentations include gastrointestinal symptoms; cutaneous reactions such as urticaria; and respiratory complaints, including wheezing and cough (22). In severe cases, these manifestations can progress to systemic involvement and culminate in life-threatening anaphylaxis (22). In clinical practice, pediatric FA severity is commonly evaluated using symptom-based scoring systems together with serum IgE measurements (23, 24). Accordingly, we assessed BFJPM efficacy using an integrated framework that combined allergic symptom scores, total serum IgE levels, and histopathological alterations in the small intestinal mucosa.

Experimental findings showed that mice receiving BFJPM-H displayed improved weight gain compared with the model group. Consistently, BFJPM-H markedly reduced allergy symptom scores and serum IgE levels, indicating attenuation of IgE-mediated hypersensitivity and overall disease severity. Among the tested doses, BFJPM-H exerted the most robust therapeutic effect. Histological assessment further supported a mucosa-protective role of BFJPM, as treatment ameliorated FA-associated intestinal injury and normalized the V/C ratio, thereby helping to preserve mucosal integrity and strengthen barrier function.

### 4.2 BFJPM altered the gut microbiota composition in OVA-induced food-allergic mice

The focus on the gut microbiota was biologically motivated by both the intestinal localization of FA and the oral administration of BFJPM. In addition, previous studies have suggested that herbal prescriptions related to the BFJPM formula are closely associated with microbiota regulation. For example, Yupingfeng Powder has been reported to modulate the upper respiratory tract microecology in mice, whereas Liujunzi Decoction can prevent antibiotic-associated intestinal dysbiosis (25, 26). Moreover, major active constituents of herbs contained in BFJPM, including *Astragalus membranaceus*, *Atractylodes macrocephala*, *Pseudostellaria heterophylla*, and *Poria cocos*, have been reported to regulate gut microecology and ameliorate gastrointestinal diseases (8–11).

In this study, 16S rRNA profiling showed that BFJPM reshaped the gut microbiota in OVA-induced FA mice, accompanied by increased ASV richness. Consistently, β-diversity analyses revealed clear separation of microbial community structures among groups, indicating BFJPM-associated remodeling of the intestinal ecosystem. Notably, the microbiota-related outcomes did not exhibit a strictly monotonic dose-response relationship. Although BFJPM-H showed the strongest therapeutic effect on allergic symptoms and serum IgE, BFJPM-L produced the most prominent improvement in α-diversity indices. This discrepancy suggests that microbiota diversity metrics and clinical anti-allergic efficacy may reflect related but distinct biological dimensions. Given the ecological complexity of the gut microbiota and the multi-component nature of BFJPM, different doses may induce distinct rather than linearly escalating microbial responses.

At the phylum level, BFJPM reshaped the microbial profile by lowering the proportions of *Firmicutes* and *Bacteroidetes* while increasing *Actinobacteria* and modulating other bacterial taxa. This pattern aligns with the dynamic microbiota changes reported in infants with milk protein allergy by Li Xinyue et al. (27). Notably, *Actinobacteria* represent a major source of antibiotic-producing microorganisms and have long been exploited for the discovery and production of clinically relevant antimicrobial agents (28). In addition to antibacterial activity, Actinobacteria-derived metabolites exhibit diverse bioactivities, including antitumor, antifungal, and antimalarial effects (28). At the genus level, BFJPM treatment led to a significant reduction in the relative abundance of *Lactobacillus*. In parallel, several genera—including *Candidatus Arthromitus*, *Streptococcus*, *Corynebacterium*, and *Staphylococcus*—showed marked enrichment. *Candidatus Arthromitus* has been reported to play an important role in intestinal immune regulation and in shaping host immune responses (29). Consistent with its immunomodulatory relevance, animal studies have shown reduced *Candidatus Arthromitus* abundance in FA models (30).

Given the superior therapeutic efficacy observed in the BFJPM-H group, we integrated LEfSe, Random Forest, and correlation analyses to identify *Saccharopolyspora* as a characteristic genus associated with this treatment group. As a member of the actinomycetes, *Saccharopolyspora* has attracted considerable interest as a potential source of antibiotics and other bioactive therapeutic agents (31, 32). Correlation analysis indicated a negative association between *Saccharopolyspora* abundance and IgE levels. This finding suggests that *Saccharopolyspora* may represent a microbiota feature associated with the anti-allergic effects of BFJPM, rather than a confirmed mechanistic mediator. Thus, *Saccharopolyspora* represents a microbiota strain of interest in the study of BFJPM’s interventional effects on FA and may serve as a potential mechanistic mediator of the herbal formula’s efficacy.

### 4.3 Potential microbiota-associated mechanisms underlying the effects of BFJPM in food allergy

Network pharmacology analysis of the BFJPM–FA axis identified eight hub targets, including INS, IL6, TNF, and IL10. These molecules may represent key nodes through which BFJPM modulates FA-related responses. KEGG enrichment suggested that BFJPM acts mainly through pathways related to anti-inflammatory regulation, antiviral immune responses, and lipid metabolism, supporting a role in counteracting FA-driven inflammatory cascades. In parallel, microbiome functional prediction showed that BFJPM-H altered the metabolic potential of the gut microbiota. The most prominent changes involved carbohydrate metabolism, metabolism of cofactors and vitamins, and lipid metabolism. Notably, *Saccharopolyspora* abundance was positively correlated with carbohydrate metabolism and metabolism of cofactors and vitamins but negatively correlated with lipid metabolism. This pattern identifies *Saccharopolyspora* as a microbial taxon associated with BFJPM-related metabolic remodeling in FA.

The African trypanosomiasis pathway was enriched in both the microbiota-based KEGG functional profiling and the BFJPM–FA KEGG analysis. This overlap suggests a potential convergence between host targets and microbiota-associated functional changes. African trypanosomiasis is characterized by parasite-driven immune evasion. Mechanistically, trypanosomes can suppress IL-2 and IL-2 receptor production through prostaglandin-mediated pathways and promote the induction of immunoregulatory cell populations, including inhibitory macrophages, MDSCs, and regulatory T cells (33). In parallel, this process is accompanied by altered cytokine production—such as IFN-γ and TNF-α—which collectively facilitates immune escape and shapes the inflammatory milieu (34, 35).

### 4.4 Limitations and future perspectives

This study has several limitations. The present work was designed to integrate therapeutic evaluation with gut microbiota profiling and network-based prediction, rather than to provide definitive mechanistic proof of microbiota-dependent immune regulation. Although BFJPM treatment was accompanied by reduced allergic severity, improved intestinal histopathology, and altered gut microbial composition, these findings support associations rather than a direct causal relationship. Microbiota remodeling may contribute to symptom improvement, but it may also occur secondarily to reduced intestinal inflammation and restoration of mucosal homeostasis. In addition, H&E staining was used to evaluate overall mucosal injury and architecture, but additional staining for allergy-associated mucosal responses, such as goblet cell hyperplasia, was not performed. Serum IgE was the primary immunological readout, whereas local intestinal cytokines, epithelial barrier markers, and immune cell populations were not further examined. Moreover, the animal experiment was conducted as a single experimental cohort with multiple biological replicates per group, without independent experimental replication. Future studies incorporating independent validation cohorts, additional mucosal immune assays, epithelial barrier assessment, metabolomic profiling, and microbiota manipulation will be required to clarify how BFJPM modulates intestinal inflammation and to determine whether gut microbiota changes play a causal role in its anti-allergic effects.

## 5 Conclusion

BFJPM alleviated allergic symptoms, reduced serum IgE levels, and improved intestinal histopathology in OVA-induced FA mice. These therapeutic effects were accompanied by remodeling of the gut microbiota, suggesting that microbiota-associated changes may be involved in the response to BFJPM. Among the altered taxa, *Saccharopolyspora* showed a negative association with serum IgE levels and may represent a microbial feature associated with BFJPM treatment. Network pharmacology further identified INS, IL6, TNF, and IL10 as major putative targets of BFJPM, while the African trypanosomiasis pathway emerged as a shared signal in both host-target and microbiota-related analyses. In addition, enrichment of the African trypanosomiasis pathway—particularly prostaglandin (PG)–related immune-evasion modules—may indicate a potential point of convergence between BFJPM-regulated host pathways and microbiota-associated functional changes. Collectively, these findings support an association among BFJPM treatment, improved allergic outcomes, and gut microbiota remodeling but do not establish a direct causal relationship. Further studies are needed to clarify the microbiota- and metabolite-related mechanisms underlying the effects of BFJPM in FA.

## Data Availability

The raw data supporting the conclusions of this article will be made available by the authors, without undue reservation.

## References

[1] Tong X Zhang JJ . Mechanism of immune sensitization and immunotherapy of food allergy. Chin J Allergy Clin Immunol. (2022) 16:636–41. doi: 10.3969/j.issn.1673-8705.2022.06.012

[2] Ma ZY Chen L Xian RL Fang HP Wang J Hu Y . Time trends of childhood food allergy in China: three cross-sectional surveys in 1999, 2009, and 2019. Pediatr Allergy Immunol Off Publ Eur Soc Pediatr Allergy Immunol. (2021) 32:1073–9. doi: 10.1111/pai.13490. PMID: 33651447

[3] Chernikova DA Zhao MY Jacobs JP . Microbiome therapeutics for food allergy. Nutrients. (2022) 14:5155. doi: 10.3390/nu14235155. PMID: 36501184 PMC9738594

[4] Wang NR Li HQ . The analysis of intestinal microflora of infants with food allergy and healthy infants. Chin J Microecol. (2006) 18:110–1. doi: 10.13381/j.cnki.cjm.2006.02.014

[5] Melli LCFL do Carmo-Rodrigues MS Araújo-Filho HB Solé D de Morais MB . Intestinal microbiota and allergic diseases: a systematic review. Allergol Immunopathol (Madr). (2016) 44:177–88. doi: 10.1016/j.aller.2015.01.013. PMID: 25985709

[6] Yang SP Zhao T Yu HY Zhou C . Research progress in the relationship between gut microbiota and food allergy in children. Acta Microbiol Sin. (2024) 64:2224–41. doi: 10.13343/j.cnki.wsxb.20230802

[7] Shu L Zhang Z Xie J Jiang P Liu KL Mo FJ . Effects of staged TCM differentiation and treatment on the serum IgE and ECP of cough variant asthma children. J TCM Univ Hunan. (2012) 32:58–60. doi: 10.3969/j.issn.1674-070X.2012.11.017.058.03

[8] Feng W Liu J Tan Y Ao H Wang J Peng C . Polysaccharides from Atractylodes macrocephala koidz. Ameliorate ulcerative colitis via extensive modification of gut microbiota and host metabolism. Food Res Int. (2020) 138:109777. doi: 10.1016/j.foodres.2020.109777. PMID: 33288163

[9] Chen T Xie L Shen M Yu Q Chen Y Xie J . Recent advances in Astragalus polysaccharides: structural characterization, bioactivities and gut microbiota modulation effects. Trends Food Sci Technol. (2024) 153:104707. doi: 10.1016/j.tifs.2024.104707. PMID: 38826717

[10] Zou Y-T Zhou J Wu C-Y Zhang W Shen H Xu J-D . Protective effects of Poria cocos and its components against cisplatin-induced intestinal injury. J Ethnopharmacol. (2021) 269:113722. doi: 10.1016/j.jep.2020.113722. PMID: 33352240

[11] Xiao Q Zhao L Jiang C Zhu Y Zhang J Hu J . Polysaccharides from pseudostellaria heterophylla modulate gut microbiota and alleviate syndrome of spleen deficiency in rats. Sci Rep. (2022) 12:20217. doi: 10.1038/s41598-022-24329-9. PMID: 36418343 PMC9684442

[12] Meng X Liu CL Chen C Xie Q Fu WH Xue WT . Exploration of gut microbiota changes in BALB/c mice sensitized with ovalbumin without immunologic adjuvant. J Food Sci Bio/Technol. (2024) 43:14–25. doi: 10.12441/spyswjs.20211202001

[13] Cárdenas-Torres FI Cabrera-Chávez F Arvizu-Flores AA Flores-Mendoza LK Lopez-Teros V Astiazaran-Garcia H . Assessment of the route of exposure to ovalbumin and cow’s milk proteins on the induction of IgE responses in BALB/c mice. Biology. (2022) 11:542. doi: 10.3390/biology11040542. PMID: 35453740 PMC9031655

[14] Comstock SS Gershwin LJ Teuber SS . Effect of walnut (juglans regia) polyphenolic compounds on ovalbumin-specific IgE induction in female BALB/c mice. Ann N Y Acad Sci. (2010) 1190:58–69. doi: 10.1111/j.1749-6632.2009.05274.x. PMID: 20388137

[15] He SL Wang J Wang JJ . TCM Reseach Desigh & Statistics. Changsha: Hunan Science Technology Press (2005). p. 395.

[16] Zhao JD . Diagnostics of chinese medical syndromes. Beijing: People’s Medical Publishing House (1987).

[17] Yandza T Tauc M Saint-Paul M-C Ouaissi M Gugenheim J Hébuterne X . The pig as a preclinical model for intestinal ischemia-reperfusion and transplantation studies. J Surg Res. (2012) 178:807–19. doi: 10.1016/j.jss.2012.07.025. PMID: 22884450

[18] Yan L Wu C-R Wang C Yang C-H Tong G-Z Tang J-G . Effect of candida albicans on intestinal ischemia-reperfusion injury in rats. Chin Med J (Engl). (2016) 129:1711–8. doi: 10.4103/0366-6999.185862. PMID: 27411459 PMC4960961

[19] Guo MF Di JX Xiao NQ Peng MJ . One of the short-chain fatty acids (SCFAs), sodium propionate, can reduce the dosage of sishen pill in regulating the intestinal microbiota in diarrhea with kidney-yang deficiency syndrome. J Inflammation Res. (2025) 18:7195–214. doi: 10.2147/JIR.S522689. PMID: 40491784 PMC12147927

[20] Guo MM Wu Y Peng MJ Xiao NQ Lei ZJ Tan ZJ . Decreasing of trimethylamine N-oxide by cecal microbiota and choline-trimethylamine lyase are associated with sishen pill on diarrhea with kidney-yang deficiency syndrome. J Inflammation Res. (2024) 17:7275–94. doi: 10.2147/JIR.S470254. PMID: 39429849 PMC11486675

[21] Xie SQ Fang LY Deng N Shen JX Tan ZJ Peng X . Targeting the gut-kidney axis in diarrhea with kidney-yang deficiency syndrome: the role of sishen pills in regulating TMAO-mediated inflammatory response. Med Sci Monit Int Med J Exp Clin Res. (2024) 30:e944185. doi: 10.12659/MSM.944185. PMID: 38898640 PMC11305074

[22] Yu W Freeland DMH Nadeau KC . Food allergy: immune mechanisms, diagnosis and immunotherapy. Nat Rev Immunol. (2016) 16:751–65. doi: 10.1038/nri.2016.111. PMID: 27795547 PMC5123910

[23] Li WY Zhou SM Wang SH Sui FX Gao WH Liu Q . Assessment of cow’s milk-related symptom scores in early identification of cow’s milk protein allergy in infants in Shenzhen: A multi-center survey analysis. J Clin Pediatr. (2020) 38:603–6. doi: 10.1186/s12887-019-1563-y. PMID: 31179927 PMC6556956

[24] Luo Y Yao X . Role and targeted therapy of IgE in allergic diseases. Chin J Allergy Clin Immunol. (2021) 15:443–7. doi: 10.3969/j.issn.1673-8705.2021.04.014

[25] Liu Q Li Q Yuan J Li J Kang L Li Z . Effects of different doses of Yuping Feng powder on microecology of upper respiratory tract in mice. China Contin Med Educ. (2017) 9:192–3. doi: 10.3969/j.issn.1674-9308.2017.24.104

[26] Su Y Zhang Z Zhong X-P Ding P . Clinical study on antibiotic associated intestinal dysbacteriosis in patients’ prevention of Liujunzi decoction. Contin Med Educ. (2021) 35:156–8. doi: 10.13343/j.cnki.wsxb.20230802

[27] Li XY Wang S Zhang H Li ZL . Characteristics of dynamic changes in the gut microbiome of infants with cow’s milk protein allergy. J Clin Pediatr. (2022) 40:831–8. doi: 10.12372/jcp.2022.21e1524

[28] Yang Y Li HL Ma KL Wang YX Niu SQ . Actinomycetes and their metabolites: visual analysis based on CiteSpace. Acta Microbiol Sin. (2022) 62:3825–43. doi: 10.13343/j.cnki.wsxb.20220088

[29] Hedblom GA Reiland HA Sylte MJ Johnson TJ Baumler DJ . Segmented filamentous bacteria - metabolism meets immunity. Front Microbiol. (2018) 9:1991. doi: 10.3389/fmicb.2018.01991. PMID: 30197636 PMC6117376

[30] Gu BH Rim CY Lee SJ Kim TY Joo SS Lee SJ . Alteration of gut immunity and microbiome in mixed granulocytic asthma. Biomedicines. (2022) 10:2946. doi: 10.3390/biomedicines10112946. PMID: 36428515 PMC9687559

[31] Frykman S Leaf T Carreras C Licari P . Precursor-directed production of erythromycin analogs by saccharopolyspora erythraea. Biotechnol Bioeng. (2001) 76:303–10. doi: 10.1002/bit.10086. PMID: 11745157

[32] Sayed AM Abdel-Wahab NM Hassan HM Abdelmohsen UR . Saccharopolyspora: an underexplored source for bioactive natural products. J Appl Microbiol. (2020) 128:314–29. doi: 10.1111/jam.14360. PMID: 31230389

[33] Darji A Beschin A Sileghem M Heremans H Brys L De Baetselier P . *In vitro* simulation of immunosuppression caused by trypanosoma brucei: active involvement of gamma interferon and tumor necrosis factor in the pathway of suppression. Infect Immun. (1996) 64:1937–43. doi: 10.1128/iai.64.6.1937-1943.1996. PMID: 8675290 PMC174019

[34] Amin DN Vodnala SK Masocha W Sun B Kristensson K Rottenberg ME . Distinct toll-like receptor signals regulate cerebral parasite load and interferon α/β and tumor necrosis factor α-dependent T-cell infiltration in the brains of trypanosoma brucei-infected mice. J Infect Dis. (2012) 205:320–32. doi: 10.1093/infdis/jir734. PMID: 22116836 PMC3244369

[35] Magez S Radwanska M Beschin A Sekikawa K De Baetselier P . Tumor necrosis factor alpha is a key mediator in the regulation of experimental trypanosoma brucei infections. Infect Immun. (1999) 67:3128–32. doi: 10.1128/IAI.67.6.3128-3132.1999. PMID: 10338530 PMC96631

