# Supplementary material for: BFJPM ameliorates OVA-induced food allergy in a murine model accompanied by gut microbiota remodeling
Source: Front Immunol. 2026 May 28;17:1827925. doi: 10.3389/fimmu.2026.1827925 (PMC13252777; doi:10.3389/fimmu.2026.1827925)
Supplement: Supplementary file 1 [file Table1.docx]

Supplementary Material

## Original H&E-stained images of small intestinal tissues


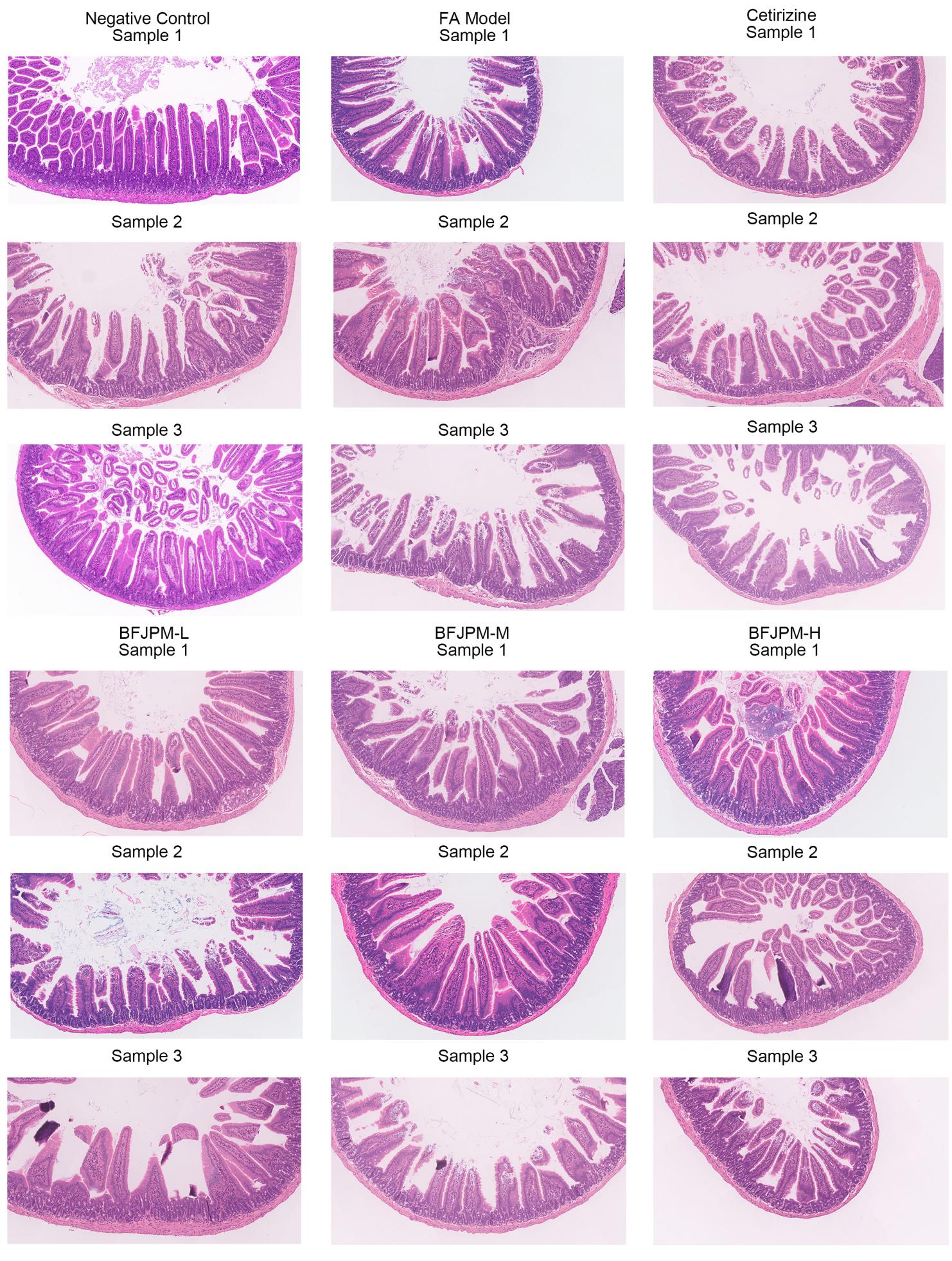


**Supplementary Figure 1.** Original hematoxylin and eosin (H&E)-stained images of small intestinal tissues corresponding to Figure 2A in the main manuscript. These images are provided as supplementary source data for the histological evaluation. A total of 18 original microscopy images are included, covering the negative control, FA model, cetirizine, BFJPM-L, BFJPM-M, and BFJPM-H groups. All images were obtained under identical staining, scanning, and image acquisition conditions. Images are shown at ×40 magnification. Scale bar = 250 μm.
